# A strategic expression method of miR-29b and its anti-fibrotic effect based on RNA-sequencing analysis

**DOI:** 10.1371/journal.pone.0244065

**Published:** 2020-12-17

**Authors:** Xiaoming Fan, Yingnyu Gao, Xiaolu Zhang, Haroon Y. Lughmani, David J. Kennedy, Steven T. Haller, Sandrine V. Pierre, Joseph I. Shapiro, Jiang Tian

**Affiliations:** 1 Department of Medicine, University of Toledo, Toledo, Ohio, United States of America; 2 Marshall Institute for Interdisciplinary Research, Marshall University, Huntington, West Virginia, United States of America; 3 Joan C. Edwards School of Medicine, Department of Biomedical Sciences, Marshall University, Huntington, West Virginia, United States of America; University of Cincinnati College of Medicine, UNITED STATES

## Abstract

Tissue fibrosis is a significant health issue associated with organ dysfunction and failure. Increased deposition of collagen and other extracellular matrix (ECM) proteins in the interstitial area is a major process in tissue fibrosis. The microRNA-29 (miR-29) family has been demonstrated as anti-fibrotic microRNAs. Our recent work showed that dysregulation of miR-29 contributes to the formation of cardiac fibrosis in animal models of uremic cardiomyopathy, whereas replenishing miR-29 attenuated cardiac fibrosis in these animals. However, excessive overexpression of miR-29 is a concern because microRNAs usually have multiple targets, which could result in unknown and unexpected side effect. In the current study, we constructed a novel Col1a1-miR-29b vector using collagen 1a1 (Col1a1) promoter, which can strategically express miR-29b-3p (miR-29b) in response to increased collagen synthesis and reach a dynamic balance between collagen and miR-29b. Our experimental results showed that in mouse embryonic fibroblasts (MEF cells) transfected with Col1a1-miR-29b vector, the miR-29b expression is about 1000 times less than that in cells transfected with CMV-miR-29b vector, which uses cytomegalovirus (CMV) as a promoter for miR-29b expression. Moreover, TGF-β treatment increased the miR-29b expression by about 20 times in cells transfected with Col1a1-miR-29b, suggesting a dynamic response to fibrotic stimulation. Western blot using cell lysates and culture media demonstrated that transfection of Col1a1-miR-29b vector significantly reduced TGF-β induced collagen synthesis and secretion, and the effect was as effective as the CMV-miR-29b vector. Using RNA-sequencing analysis, we found that 249 genes were significantly altered (180 upregulated and 69 downregulated, at least 2-fold change and adjusted p-value <0.05) after TGF-β treatment in MEF cells transfected with empty vector. The Kyoto Encyclopedia of Genes and Genomes (KEGG) pathway analysis using GAGE R-package showed that the top 5 upregulated pathways after TGF-β treatment were mostly fibrosis-related, including focal adhesion, ECM reaction, and TGF-β signaling pathways. As expected, transfection of Col1a1-miR-29b or CMV-miR-29b vector partially reversed the activation of these pathways. We also analyzed the expression pattern of the top 100 miR-29b targeting genes in these cells using the RNA-sequencing data. We identified that miR-29b targeted a broad spectrum of ECM genes, but the inhibition effect is mostly moderate. In summary, our work demonstrated that the Col1a1-miR-29b vector can be used as a dynamic regulator of collagen and other ECM protein expression in response to fibrotic stimulation, which could potentially reduce unnecessary side effect due to excessive miR-29b levels while remaining an effective potential therapeutic approach for fibrosis.

## Introduction

Formation of tissue fibrosis is a driving force for disease progression that leads to worsened organ function and failure [1, 2]. Cardiac fibrosis plays an important role in etiology of almost all forms of heart failure and particularly in heart failure with preserved ejection fraction [3, 4]. Myocardial infarction (MI) is a major cause of cardiac death and massive cardiac tissue fibrosis. There are two phases of tissue fibrosis after MI [5]. The initial formation of a fibrotic scar at the infarcted area is critical to prevent the heart from rupture, which is defined as reparative fibrosis. The second phase of fibrosis is observed in the remote non-infarcted area, often referred to as reactive fibrosis or interstitial fibrosis. The interstitial fibrosis in the non-ischemic area could significantly deteriorate heart function post MI. Severe cardiac fibrosis can lead to sudden cardiac death even in those without cardiac symptoms or ischemic injury [6–8], while reducing cardiac fibrosis in clinical and animal models has been shown to improve cardiac function [9–12].

Since increased synthesis and secretion of collagen and other extracellular matrix (ECM) proteins is a critical step in fibrosis formation [2, 13–16], directly targeting the ECM protein synthesis by microRNA (miR) has become a potential therapeutic strategy for tissue fibrosis [17]. Among these, miRNA-29 family microRNAs have been demonstrated as anti-fibrotic in different animal models with tissue fibrosis [18–23]. The miR-29 family includes miR-29a, miR-29b, and miR-29c. In humans, the miR-29b-1 and miR-29a are clustered on chromosome 7, while miR-29b-2 and miR-29c are located on chromosome 1 [24]. The sequence of miR-29b-1 and miR-29b-2 are identical, and the targets of the three miR-29 family members are also similar.

Our recent work [25–27] has demonstrated that Na/K-ATPase signaling downregulates miR-29b expression through Src/NFκB pathway and induces collagen synthesis and tissue fibrosis. We also showed that transfection of miR-29b mimics or restoring endogenous miR-29b by blocking Na/K-ATPase signaling using pNaKtide, a Src inhibiting peptide developed in our laboratory, inhibited collagen synthesis in primary cultured cardiac fibroblasts and attenuated cardiac fibrosis in animal models of uremic cardiomyopathy [25, 28]. However, due to its multi-target effect, an excessive amount of miR-29b could induce unexpected side effects such as aortic dilation and aneurysm [29]. In the current project, we constructed an expression vector that can strategically express miR-29b using a Col1a1 promoter to dynamically regulate collagen synthesis in response to fibrotic stimulation, which will result in the lower basal level of miR-29b, while remaining a potential therapeutic for fibrosis.

## Materials and methods

### Animals

Animal experiments were conducted in accordance with the National Institutes of Health, Guide for the Care and Use of Laboratory Animals under protocols (IACUC# 108917) approved by the Institutional Animal Care and Use Committee at the University of Toledo. Adult male and female C57BL/6 mice at 2–3 months of age were used for this study. All mice were reared under a 12 h dark/light cycle, fed with standard chow, and provided with water ad libitum. These conditions were utilized for the entire duration of the experiment. Animals were anesthetized with 2% isoflurane during experiments to reduce the suffering and euthanized by injecting ketamine/xylazine (500/50 mg/kg).

### Selection of Collagen 1a1 (Col1a1) promoter and construction of Col1a1-luciferase and Col1a1-EGFP plasmid vectors

To screen a Col1a1 promoter that is responsive to fibrotic stimulation, we cloned several Col1a1 promoter sequences into a pcDNA3 plasmid with luciferase (Col1a1-luc). Based on this luciferase assay result, we selected a rat Col1a1 promoter for the construction of the Col1a1-miR-29b vector. To test the in vivo delivery of the vector, we engineered a Col1a1-EGFP and a CMV-RFP into the pcDNA3 plasmid, in which the EGFP serves as a marker for activation of Col1a1 promoter, while the RFP serves as a marker for transfection efficiency.

### Construction of Col1a1-miR29b CMV-miR-29b and plasmid vectors

To construct the miR-29b overexpression vector (Col1a1-miR-29b), a pcDNA3 plasmid was used as a backbone for the construction of miR-29b expression vectors. First, an EGFP sequence and human miR-29b-1 sequence was engineered into the downstream of the Col1a1 promoter in the pcDNA3 plasmid. The miR-29b gene sequence was cloned from a pcDNA3-miR29b plasmid (Addgene, Plasmid No.: 21121). An mRFP1 sequence was inserted under the SV40 promoter in the same plasmid as a transfection marker. To construct the CMV-miR-29b expression vector, we replaced the Col1a1 promoter with a CMV promoter and all other structure of the plasmid was kept the same. This CMV promoted miR-29b expression vector (CMV-miR-29b) serves as a positive control in all experiments. An empty pcDNA3 vector was used as a negative control.

### Cell treatment and plasmid transfection

Mouse Embryonic Fibroblasts (MEF) were obtained from American Type Culture Collection and maintained in Dulbecco's modified Eagle's medium (DMEM) supplemented with 10% fetal bovine serum, 100 units/ml penicillin, and 100 μg/ml streptomycin in 5% CO2 humidified incubator. MEF cells grown on 12-well plates were transfected with 1 pmol DNA/ml media (about 3 μg/ml for CMV-miR29b and 5 μg/ml for Col1a1-miR29b based on the vector size) using Lipofectamine 3000 reagent from ThermoFisher Scientific (Cat. No.: L3000008) following the manufacturer's protocol. Empty pcDNA3 vector-transfected cells were used as non-treatment control. At 6 h post-transfection, MEF cells were treated with TGF-β (R&D systems, Cat. No.: 240-B) for an additional 24 h. Cells without TGF-β treatment were used as non-treatment control. Cell lysates were then collected for Western blot or RNA extraction at the end of the experiment. Culture media were also collected for the measurement of secreted collagen.

### Lentivirus packaging and transfection

The plasmid with Col1a1-EGFP and CMV-RFP was packed into lentiviruses by a commercial company (VectorBuilder Inc. at Chicago, IL). For MEF cells transfection, purified lentivirus (~10^9^ TU/ml) was premixed with polybrene (final concentration of 8 μg/ml) and added to cultured MEF cells in a 6-well plate at 60 μl of the virus suspension per well. Cells were cultured for an additional 48h and the fluorescence of EGFP and RFP were examined under a fluorescent microscope. For *in vivo* delivery, experimental mice were anesthetized with 2% isoflurane, and about 100 μl of lentivirus (10^9^ TU/ml) mixed with polybrene was instilled to the lung surface of C57BL/6 mice by the intra-trachea method. On the 7^th^ day after the delivery, mice were euthanized by injection of ketamine/xylazine (500/50 mg/kg bodyweight), and the lung tissue was collected into the OTC-containing chamber for preparation of frozen tissue sections. The EGFP and RFP fluorescence were imaged using a Leica confocal microscope.

### Western blot

Cell lysates collected in ice-cold Radioimmunoprecipitation (RIPA) buffer (Santa Cruz Biotechnology, Cat No.: sc-24948) containing 2 mM PMSF, 1% protease inhibitor cocktail, and 1 mM sodium orthovanadate were centrifuged at 14,000 g for 15 min, and the supernatants were used for Western blot. Equal protein amount of cell lysates or an equal volume of cell culture medium were separated on an SDS-PAGE gel, and Western blot was performed to probe for type I collagen using an anti-collagen primary antibody (SouthernBiotech, Cat. No.: 1310–01). GAPDH (Santa Cruz Biotechnology, Cat. No.: sc-25778) was used as a loading control for Western blots using cell lysates.

### Quantitative RT-PCR

Total RNA was extracted from cell lysates using the miRNeasy Mini Kit (Qiagen Inc., Cat No.: 217004). The cDNAs for mRNA were synthesized using the RT^2^ First Strand cDNA synthesis kit (Qiagen Inc., Cat No.: 330404). Gene expression of Col1a1 was measured using RT2 SYBR Green qPCR reaction mix (Qiagen Inc., Cat. No.: 330501) on a Qiagen Rotor Gene Q Cycler with specific primers (Forward: CATTGTGTATGCAGCTGACTTC; and reverse: CGCAAAGAGTCTACATGTCTAGG). GAPDH (primers from Qiagen Inc., Cat No.: PPM02946E) was used as a reference. The expression level was expressed as fold change calculated by 2 ^-ΔΔCT^.

### RNA sequencing of MEF cells transfected with miR-29b expression vectors with or without TGF-β treatment

Extracted total RNA from transfected cells (with or without TGF-β treatment) was used for RNA-sequencing. RNA integrity was checked with Agilent Technologies 2100 Bioanalyzer. RNA-Sequencing was performed by LC Sciences (Houston, Texas) following their standard protocol. Briefly, Poly(A) tail-containing mRNAs were purified using oligo-(dT) magnetic beads with two rounds of purification. After purification, poly(A) RNA was fragmented using divalent cation buffer in elevated temperature. The DNA library construction is shown in the following workflow. Quality control analysis and quantification of the sequencing library were performed using Agilent Technologies 2100 Bioanalyzer High Sensitivity DNA Chip. Paired-ended sequencing was performed on Illumina’s NovaSeq 6000 sequencing system. Cutadapt and Perl scripts in the house were used to remove the reads that contained adaptor contamination, low quality bases and undetermined bases. Sequence quality was verified using FastQC (http://www.bioinformatics.babraham.ac.uk/projects/fastqc/). HISAT2 was used to map reads to the genome of ftp://ftp.ensembl.org/pub/release-96/fasta/mus_musculus/dna/. The mapped reads of each sample were assembled using StringTie, and all transcriptomes were merged to reconstruct a comprehensive transcriptome using Perl scripts and gffcompare.

### Pathway analysis based on RNA-sequencing data

The normalized count data obtained from RNA-sequencing was first analyzed using DESeq2 [30] to create a table of differentially expressed gene (DEG) list, which was then used for pathway analysis using GAGE R-package [31]. The gene mapping on specific signaling pathways was done using Pathview R-package [32].

**Statistics.** Data were presented as Mean ± SEM. Statistical analysis for Western blot and RT-qPCR were performed using Student's *t* test or One-way *ANOVA* where appropriate. Significance was accepted at p < 0.05. For RNA-sequencing analysis, the statistical analysis followed the default settings from the software packages.

## Results

### Construction of a strategic expression vector using the Col1a1 promoter that is responsive to fibrotic stimulation

**Fig 1:**
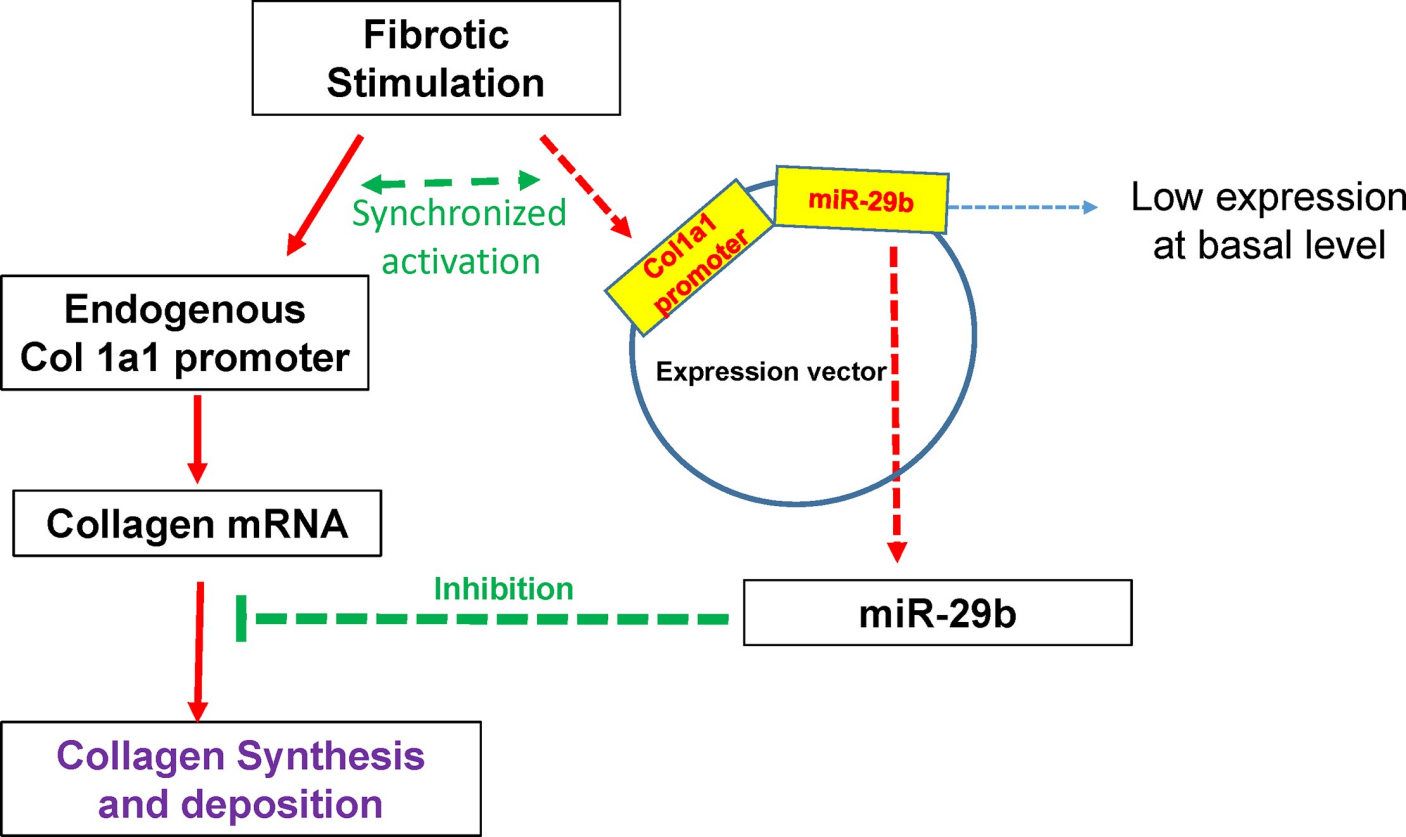
Dynamic regulation of collagen by the col1a1-miR-29b vector. The col1a1 promoter works as a sensor for collagen expression that drives the expression of miR-29b, and therefore dynamically regulates collagen production and minimizes the off-target effect of miR-29b.

As shown in Fig 1, the rational to construct a Col1a1-miR-29b vector is that fibrotic stimuli can simultaneously activate the endogenous Col1a1 promoter as well as the exogenous Col1a1 promoter. The exogenous Col1a1 promoter will then stimulate the expression of miR-29b and contra-regulate the elevated collagen expression to reach a dynamic balance between miR-29b and collagen mRNA.

**Fig 2:**
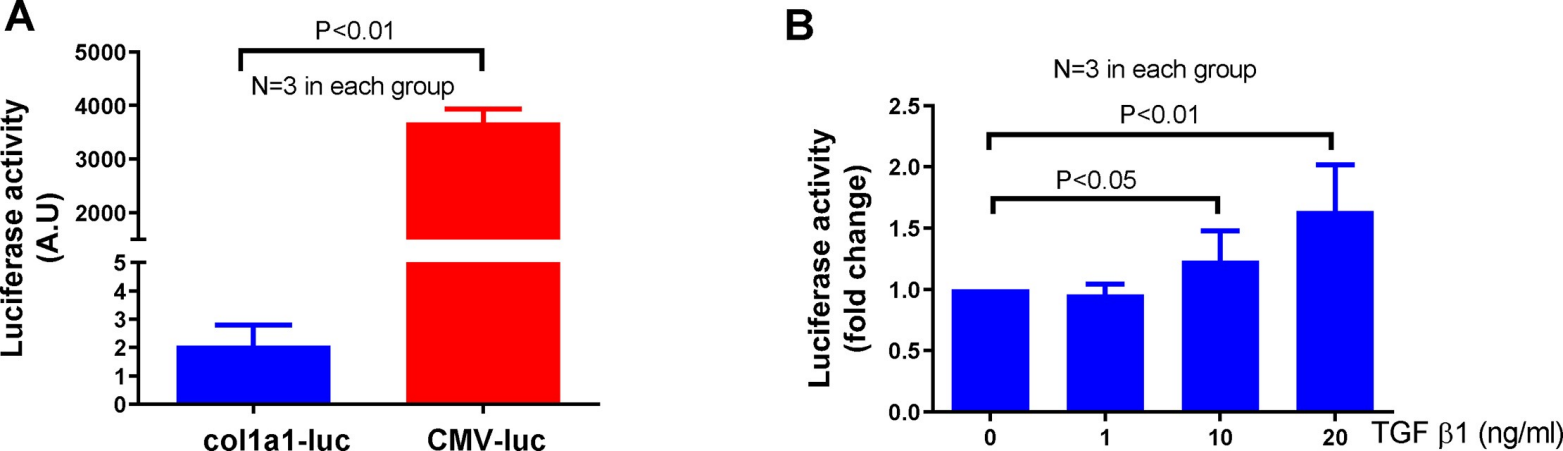
Comparison of col1a1 and CMV promoter’s regulation of downstream gene expression. (A) Both col1a1-luc and CMV-luc vectors were transfected into MEF cells for 24h. Luciferase activity was measured using a firefly luciferase substrate from Promega. (B) MEF cells transfected with col1a1-luc vector for 24 h were treated with TGF-β1 for an additional 24h and luciferase activity was measured. Each treatment was repeated 3 times (N = 3).

To test this concept, we first constructed a Col1a1-luciferase vector as described in the Method section and used CMV-luciferase vector (CMV-luc) as a control. To examine the basal luciferase expression, MEF cells were transfected with the Col1a1-luc or CMV-luc vectors for 24 h and a luciferase activity assay was then performed. As shown in Fig 2A, the luciferase activity was about 2000 times lower in Col1a1-luc transfected cells compared to cells transfected with the CMV-luc vector. To test if the Col1a1 promoter is regulated by fibrotic stimuli, MEF cells transfected with Col1a1-luc were treated with a recombinant human TGF-β1 for 24 h. The results showed that luciferase activity increased in a dose-dependent manner in response to TGF-β1 treatment (Fig 2B).

### Delivery of the expression vector in cells and animals using lentivirus

**Fig 3:**
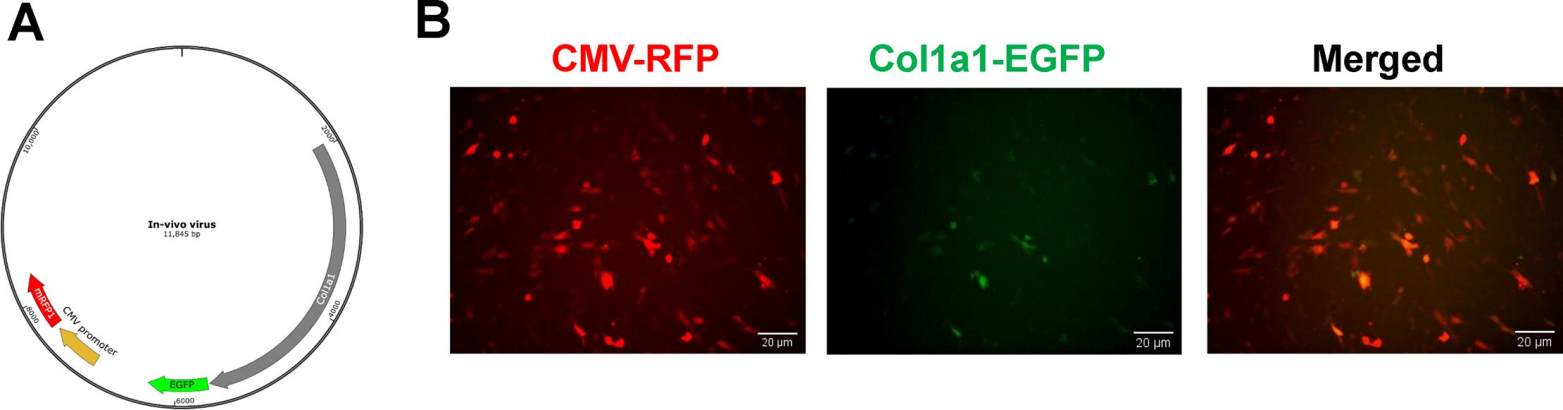
Infection by lentiviral constructs in Mouse Embryonic Fibroblast (MEF) cells. (A) The CMV-RFP/col1a1-EGFP vector was packed into lentivirus and mixed with polybrene. (B) MEF cells were cultured on a 6-well plate for 24h and infected with the lentiviruses for 48h. EGFP and RFP were visualized under a fluorescence microscope with a 20x lens.

To test if lentivirus can be used for delivery of the expression vector, we constructed a vector that contains Col1a1-EGFP and CMV-RFP shown in Fig 3A. The lentivirus packed vector was added to MEF cells cultured in a 6-well plate for 48 h. The fluorescence of EGFP and RFP were examined under a fluorescent microscope. As shown in Fig 3B, the infected MEF cells expressed very strong red RFP fluorescent signals but a very weak green EGFP signal, suggesting that the Col1a1 promoter causes a much lower expression of the downstream gene compared to the CMV promoter.

**Fig 4:**
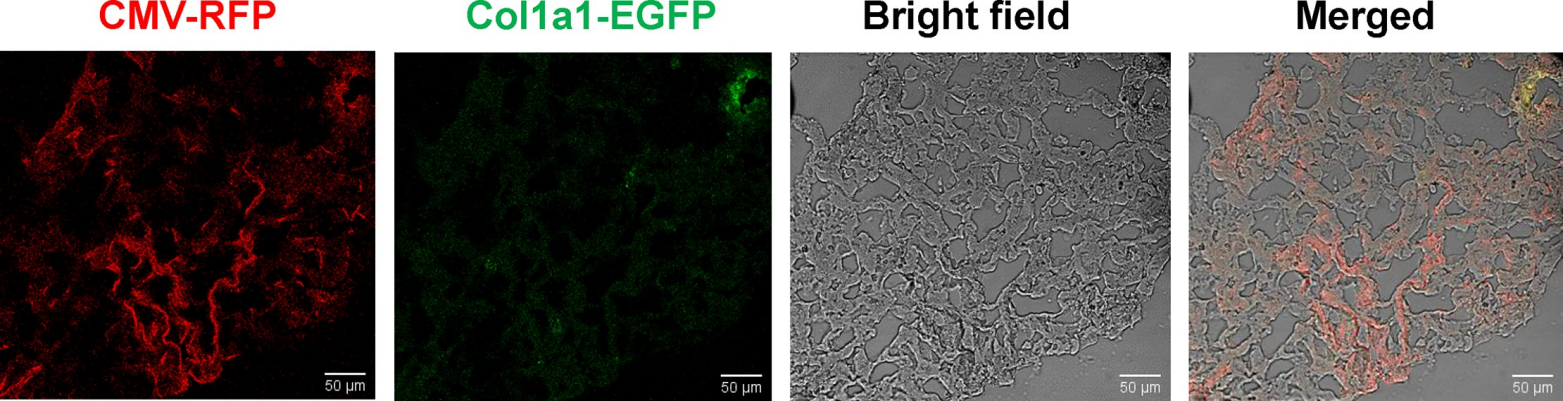
Infection of lentiviruses in Mouse Embryonic Fibroblast (MEF) cells in the lung tissue of mice. The vector was packed into lentivirus and mixed with polybrene. About 100 μl of the mixture was delivered to the lung surface by the intra-trachea method. One week after the delivery, mice were euthanized and a frozen section of lung tissue was prepared. EGFP and RFP were visualized under a Leica confocal microscope.

To test the delivery of these viruses *in vivo*, 100 μl of the virus suspension was instilled into the lung surface of C57BL/6 mice by the intra-trachea method. The lung tissue was frozen in OTC containing chamber and tissue slides were prepared under frozen conditions. EGFP and RFP fluorescent signals were imaged using a Leica confocal microscope. As shown in Fig 4, the red RFP signal was strong and could be detected in a large area of the lung tissue, but the EGFP signal was barely detected in the tissue slides. This result suggested that the Col1a1 promoter results in much less basal level gene expression in *vivo* just like in cultured cells.

### Anti-fibrotic effect of Col1a1-miR-29b and CMV-miR-29b vectors

**Fig 5:**
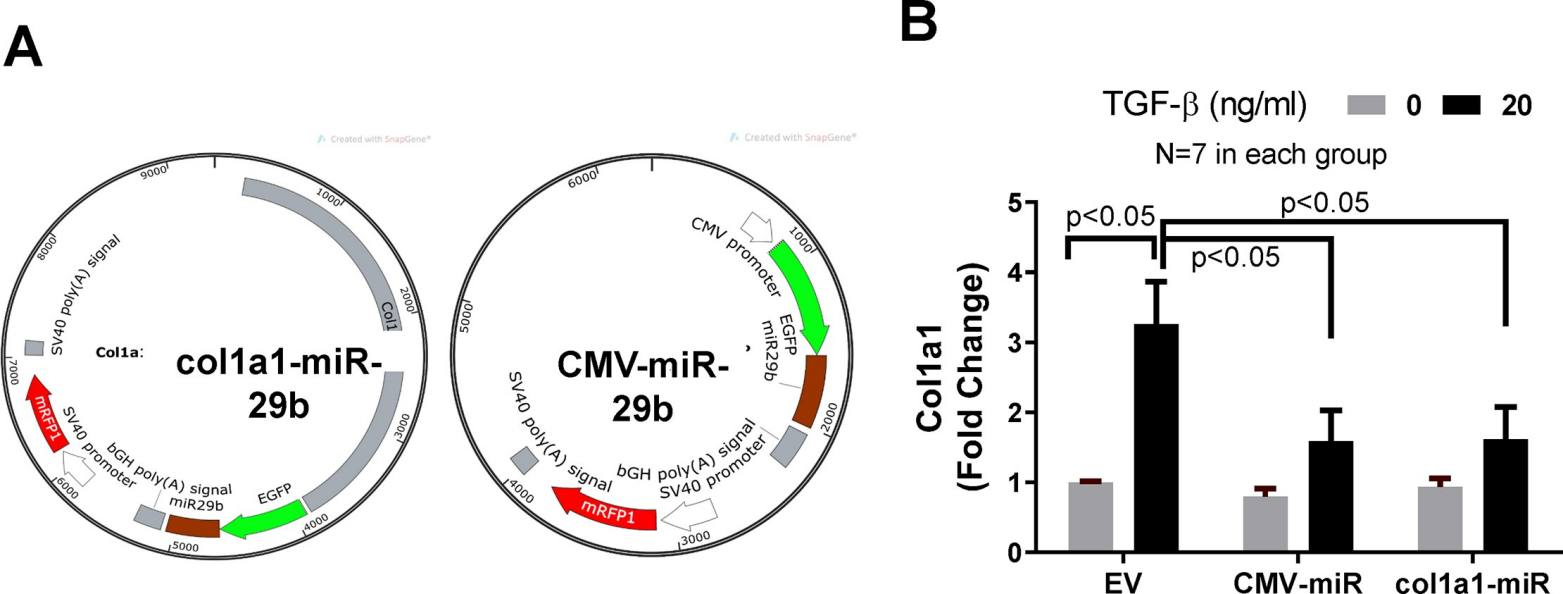
The effect of col1a1-miR-29b and CMV-miR-29b on collagen expression. (A) Structure of Col1a1-miR-29b and CMV-miR-29b vectors. (B) Collagen expression. Col1a1-miR-29b and CMV-miR-29b vectors were transfected into MEF cells for 24h before treatment with TGF-β1 for an additional 24h. Total RNA was then extracted from the cell lysates. RT-qPCR was used to measure the expression of collagen. N = 7 for each group.

**Fig 6:**
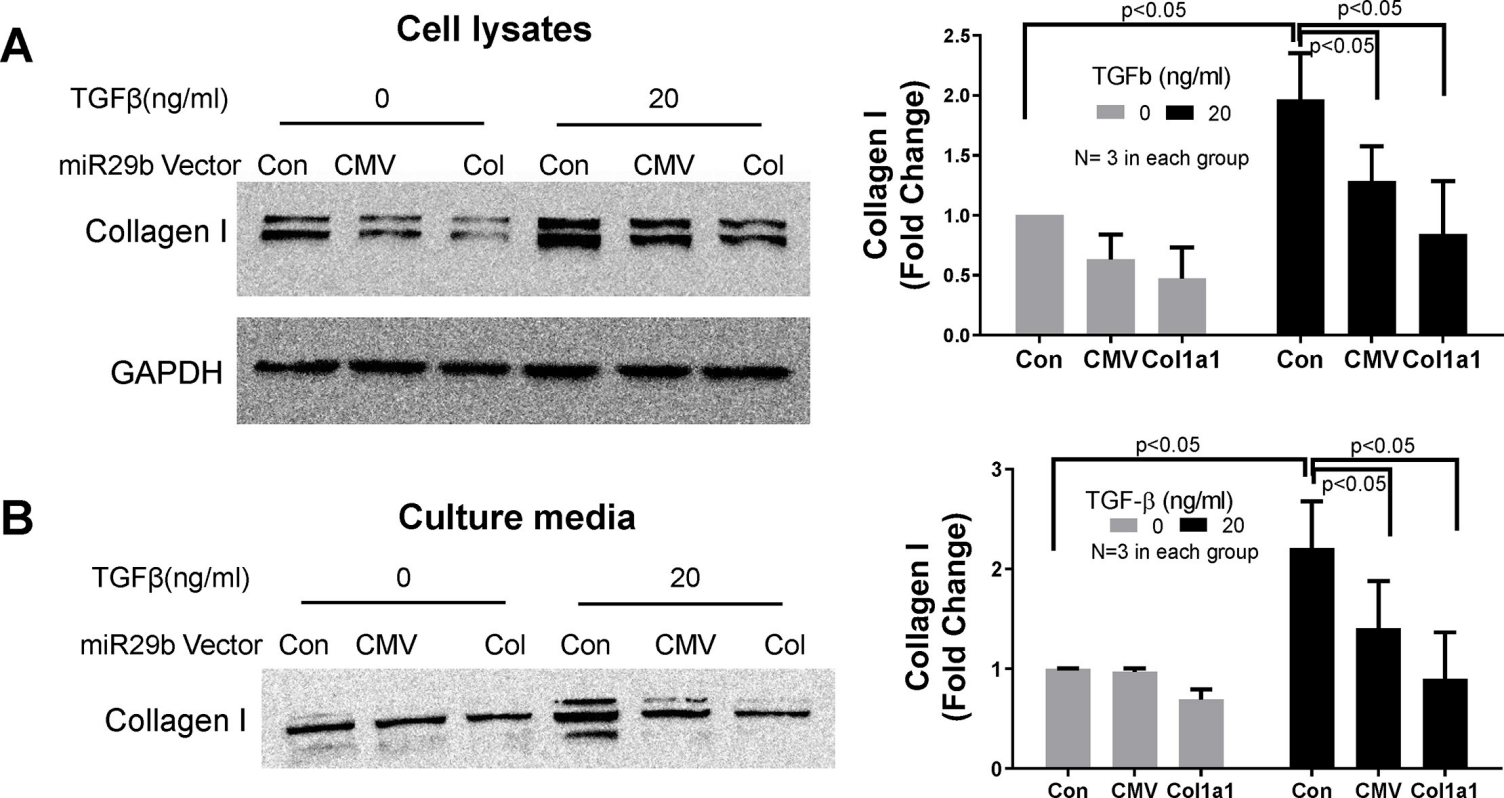
The effect of col1a1-miR-29b and CMV-miR-29b on collagen synthesis. The col1a1-miR-29b and CMV-miR-29b vectors were transfected into MEF cells for 24h before treatment with TGF-β1 for an additional 24h. Cell lysates (A) and culture medium (B) were collected for Western blot. Each of the above experiments were repeated 3 times (N = 3).

The structures of miR-29b expression vectors are shown in Fig 5A. To test their anti-fibrotic effect, MEF cells were transfected with these miR-29b expression vectors for 6 h before treatment with TGF-β (20 ng/ml) for an additional 24 h. The empty pcDNA3 vector was used as vector control. Cell lysates were collected in Trizol for RNA extraction and RT-PCR analysis. As shown in Fig 5B, treatment of TGF-β1 caused a 3-fold increase in collagen expression in control cells, while transfection of Col1a1-miR-29b or CMV-miR-29b significantly blocked the TGF-β1-induced increase in collagen expression by about 50%. To examine the protein level of collagen type I, the same experiment was conducted, and cell lysates were collected in RIPA buffer for Western blot. As shown in Fig 6A, both Col1a1-miR-29b and CMV-miR-29b vector transfection significantly blocked the TGF-β-induced increase in collagen synthesis in MEF cells. In addition, the culture media was collected from these experiments and equal volume (30 μl) of the culture media was used for Western blot to detect collagen amount. Since there has not been a commonly recognized marker for culture medium, we used equal volume for loading control. As shown in Fig 6B, the transfection of miR-29b expression vectors significantly reduced the amount of collagen secreted into the culture medium. These results demonstrated that overexpression of miR-29b can effectively block TGF-β-induced excessive collagen synthesis and secretion. The uncropped and unadjusted images of these Western blot are presented in the S1 File.

### Assessment of gene profiling change in Col1a1-miR-29b and CMV-miR-29b transfected cells using RNA sequencing

**Fig 7:**
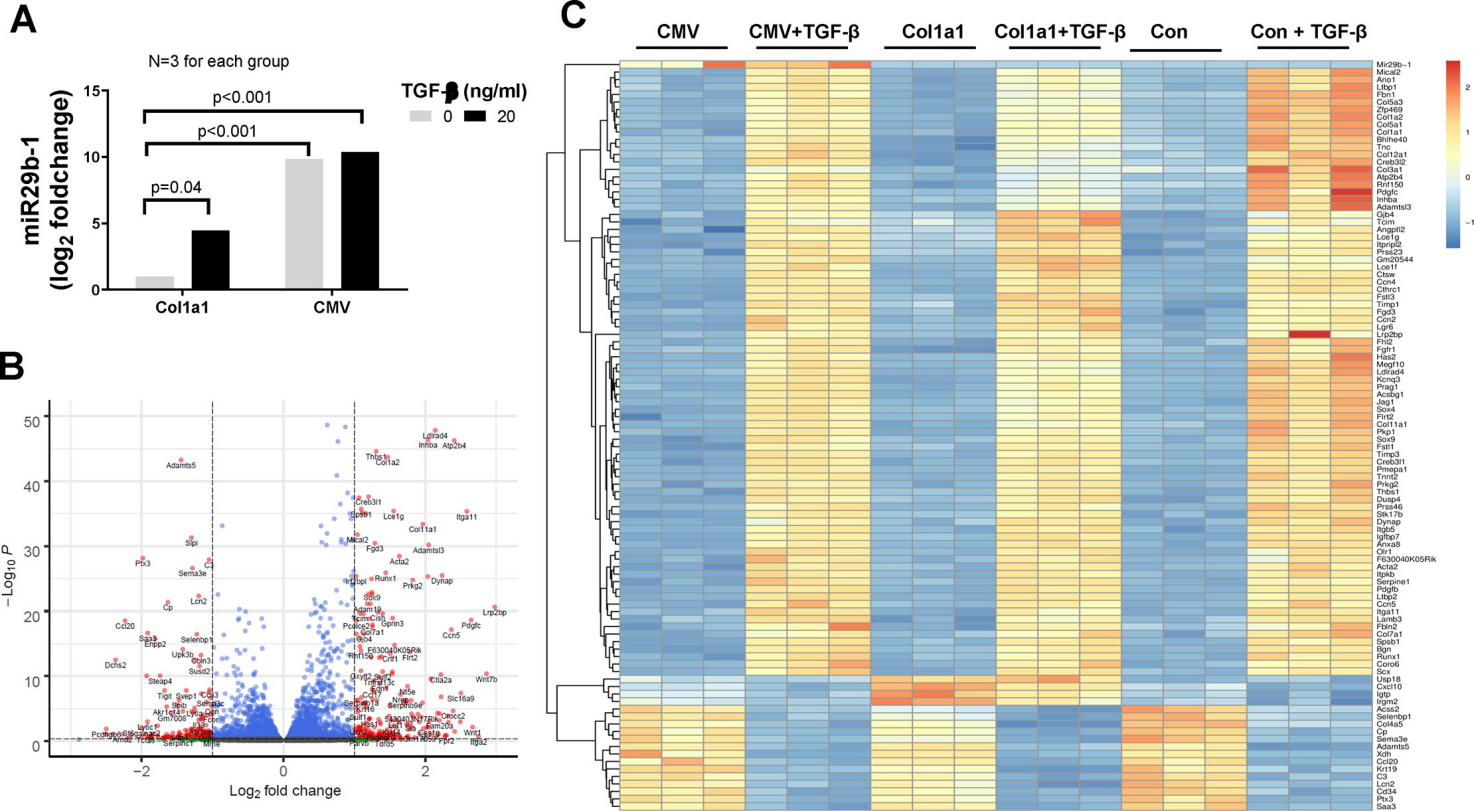
Differentially Expressed Gene (DEG) profiling in MEF cells. MEF cells transfected with empty vector, col1a1-miR-29b vector, or CMV-miR-29b vector, then treated with 20 ng/ml TGF-β for 24h. The total RNA was extracted for RNA-sequencing by LC Science. (A) The log2 foldchange of miR-29b expression cells in transfected cells. The log2 fold change and p-value were calculated using R-package DESeq2 as described in the Method section. (B) Volcano plot of significantly regulated genes after TGF-β treatment. (C) Heatmap of DEG in different groups.

To assess the gene profiling change, MEF cells were transfected with empty vector, Col1a1-miR-29b vector, or CMV-miR-29b vector for 6 h, and was then treated with TGF-β1 for an additional 24 h. The cell lysates were collected for total RNA extraction. The extracted RNA from each group were sent to LC Sciences (Houston TX) for RNA sequencing. Three repeated experiments were performed for each group. DESeq2 R-package was used to obtain the differentially expressed genes (DEG) data and FPKM data. As shown in Fig 7A, the miR-29b expression in CMV-miR-29b transfected cells is about 1000 times higher (log2 foldchange = 9.84) than that with Col1a1-miR-29b transfection in cells without TGF-β treatment. Moreover, TGF-β treatment significantly increased miR-29b expression by about 20 times or a log2 foldchange of 4.5 in Col1a1-miR-29b vector-transfected cells. TGF-β treatment did not cause a significant increase in miR-29b expression in CMV-miR-29b vector-transfected cells. These data further demonstrated the dynamic expression of miR-29b with the Col1a1 promoter in response to fibrotic regulation. In addition to miR-29b change, we identified 249 significantly regulated genes (180 upregulated and 69 downregulated, at least 2-fold change and adjusted p-value <0.05) after TGF-β treatment, which is shown in the volcano plot (Fig 7B). Using FPKM data resulted from DESeq2 analysis, a heatmap was created to show the overall gene expression change between the control and treated groups (Fig 7C). We observed that a variety of fibrosis-related genes such as different isoforms of collagen and other ECM genes were elevated in TGF-β treated groups, but overexpression of miR-29b inhibited the effect of TGF-β in these cells. TGF-β also increased the integrin alpha 2 (Itga2) to about 7-fold and increased α-SMA (Acta2) gene expression to about 3-fold. Both are closely related with tissue fibrosis. However, they were not inhibited by the miR-29b overexpression. In addition to these fibrosis-related genes, TGF-β also regulated metabolism-related gene, e.g., it significantly increased acetyl-Coenzyme A acyltransferase 1B (acaa1b) and oxidized low density lipoprotein receptor 1 (olr1), and significantly decreased arachidonate 8-lipoxygenase (Alox8) and glycine C-acetyltransferase (Gcat). The interaction of these genes and their specific physiological and pathological effect are hard to explain by individual gene expression. Therefore, we performed a KEGG pathway analysis to understand the overall effect of TGF-β treatment.

**Fig 8:**
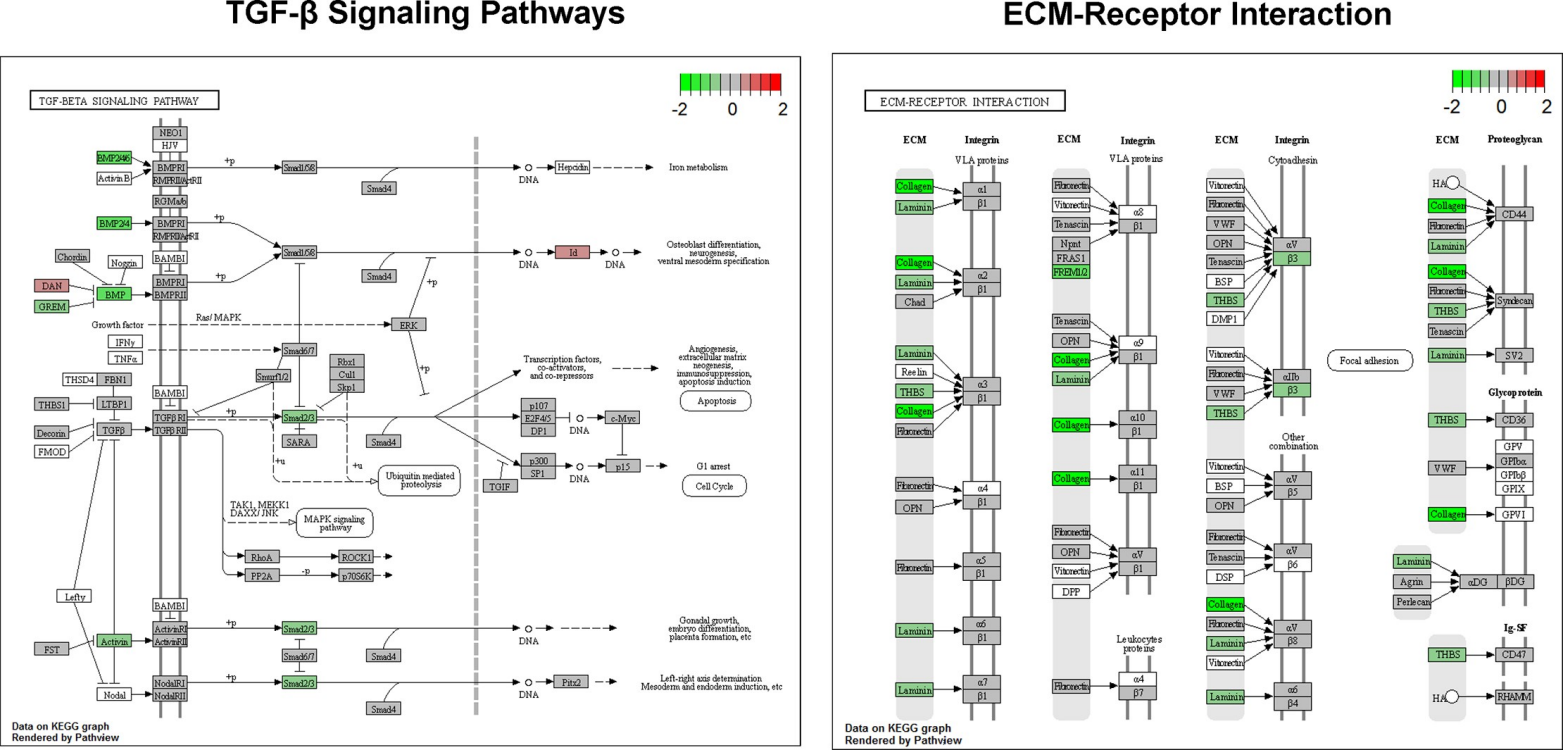
Representative gene map of upregulated KEGG pathways after TGF-β treatment. Pathway analysis was performed using GAGE R-package and a gene map was obtained using Pathview R-package as described in the Method section.

KEGG pathway analysis using GAGE R-package showed that TGF-β in empty vector-transfected cells induced upregulation of several fibrosis-related pathways including focal adhesion, ECM receptor interaction, and TGF-β pathways. The top 10 up and down regulated pathways are listed in Table 1. The representative gene maps of the upregulated KEGG pathways (TGF-β signaling pathway and ECM-receptor interaction) are shown in Fig 8. To examine the effect of miR-29b on TGF-β-activated pathways, we analyzed the pathway changes in CMV-miR-29b or Col1a1-miR-29b transfected cells versus the empty vector-transfected cells, and the result showed that TGF-β-induced focal adhesion and ECM receptor interaction pathways were downregulated by miR-29b overexpression. The pathways affected by Col1a1-miR-29b and CMV-miR-29b transfection are listed in Table 2.

**Table 1:** TGF-β-induced KEGG pathway changes in MEF cells.

Upregulated KEGG pathways	p.geomean	p.val	q.val	set.size
mmu04510 Focal adhesion	0.001	0.001	0.130	165
mmu04512 ECM-receptor interaction	0.003	0.003	0.214	66
mmu04974 Protein digestion and absorption	0.007	0.007	0.357	39
mmu04540 Gap junction	0.014	0.014	0.563	63
mmu04350 TGF-beta signaling pathway	0.061	0.061	0.964	64
mmu04370 VEGF signaling pathway	0.077	0.077	0.964	59
mmu04115 p53 signaling pathway	0.083	0.083	0.964	62
mmu00532 Glycosaminoglycan biosynthesis—chondroitin sulfate	0.106	0.106	0.964	15
mmu04810 Regulation of actin cytoskeleton	0.112	0.112	0.964	164
mmu04310 Wnt signaling pathway	0.142	0.142	0.964	117
**Downregulated KEGG pathways**	**p.geomean**	**p.val**	**q.val**	**set.size**
mmu00980 Metabolism of xenobiotics by cytochrome P450	0.003	0.003	0.309	31
mmu04612 Antigen processing and presentation	0.004	0.004	0.309	52
mmu00982 Drug metabolism—cytochrome P450	0.006	0.006	0.309	31
mmu00480 Glutathione metabolism	0.009	0.009	0.368	41
mmu00100 Steroid biosynthesis	0.024	0.024	0.627	16
mmu00620 Pyruvate metabolism	0.027	0.027	0.627	34
mmu00900 Terpenoid backbone biosynthesis	0.033	0.033	0.627	13
mmu00860 Porphyrin and chlorophyll metabolism	0.035	0.035	0.627	23
mmu04514 Cell adhesion molecules (CAMs)	0.036	0.036	0.627	68
mmu00380 Tryptophan metabolism	0.062	0.062	0.832	26

**Table 2:** Downregulated KEGG pathways by Col1a1-miR-29b and CMV-miR-29b in MEF cells treated with TGF-β.

Col1a1-miR-29b Downregulated	p.geomean	p.val	q.val	set.size
mmu04510 Focal adhesion	0.002	0.002	0.245	165
mmu04530 Tight junction	0.015	0.015	0.579	89
mmu04120 Ubiquitin mediated proteolysis	0.026	0.026	0.579	129
mmu00564 Glycerophospholipid metabolism	0.029	0.029	0.579	65
mmu04520 Adherens junction	0.030	0.030	0.579	65
mmu04666 Fc gamma R-mediated phagocytosis	0.043	0.043	0.579	71
mmu00970 Aminoacyl-tRNA biosynthesis	0.055	0.055	0.579	42
mmu04664 Fc epsilon RI signaling pathway	0.055	0.055	0.579	53
mmu01040 Biosynthesis of unsaturated fatty acids	0.056	0.056	0.579	20
mmu04150 mTOR signaling pathway	0.059	0.059	0.579	47
**CMV-miR-29b Downregulated**	**p.geomean**	**p.val**	**q.val**	**set.size**
mmu04510 Focal adhesion	0.001	0.001	0.066	160
mmu04512 ECM-receptor interaction	0.001	0.001	0.066	61
mmu04974 Protein digestion and absorption	0.011	0.011	0.546	36
mmu04540 Gap junction	0.034	0.034	0.796	63
mmu04350 TGF-beta signaling pathway	0.038	0.038	0.796	61
mmu04120 Ubiquitin mediated proteolysis	0.049	0.049	0.796	129
mmu04370 VEGF signaling pathway	0.063	0.063	0.796	58
mmu04520 Adherens junction	0.066	0.066	0.796	64
mmu04710 Circadian rhythm—mammal	0.086	0.086	0.796	20
mmu04012 ErbB signaling pathway	0.089	0.089	0.796	75

**Fig 9:**
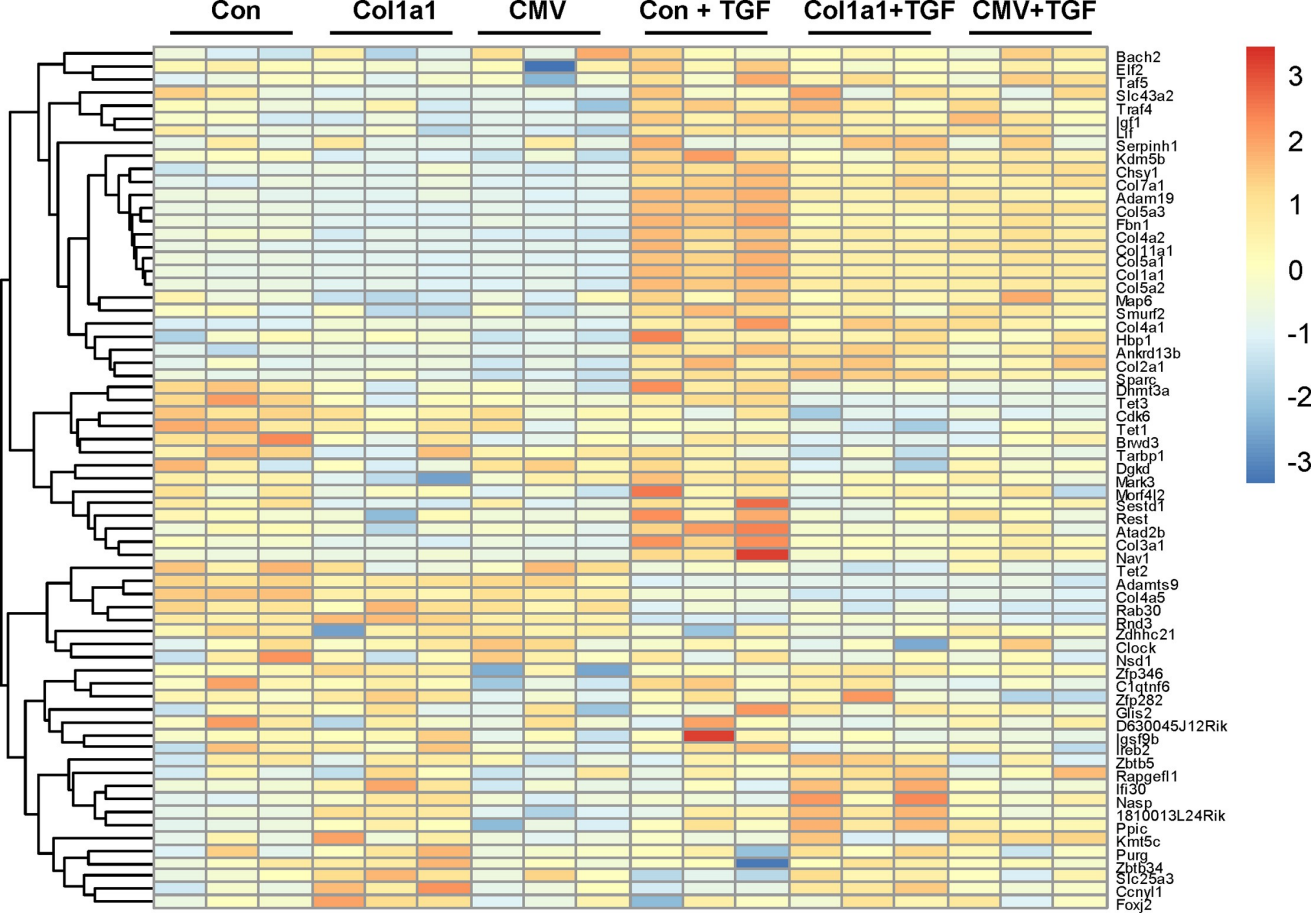
miR-29b targeting gene expression profiling after miR-29b overexpression. The FPKM value of gene expression was used for the heatmap. The top 100 miR-29b targeting gene lists were obtained from the miRDB database.

It is known that miR-29b is a potent anti-fibrotic microRNA and its predicted targeting genes include multiple ECM genes and hundreds of other genes. To examine the effect of miR-29b overexpression on its targeting genes in these cells, we picked the top 100 predicted miR-29b targeting genes based on their scores from miRDB database (http://mirdb.org/). The targeting genes’ names were matched with their differential gene expression data from RNA-sequencing. A heatmap using the FPKM data for these targeting genes was created and shown in Fig 9. We found that a broad spectrum of ECM genes was targeted by the miR-29b overexpression, but not all predicted miR-29b targeting genes were inhibited by miR-29b and some were even upregulated. However, the regulatory effect was mostly moderate. Overexpression of miR-29b decreased at least 8 isoforms of collagen gene with adjusted p-value less than 0.05, but the percentage change was between 17% (col4a2) and 45% (col3a1) in col1a1-miR-29b transfected MEF cells. In CMV-miR-29b transfected cells, the result was very similar with 9 isoforms of collagen gene inhibited. On the other hand, transfection of col1a1-miR-29b caused about 95% inhibition of small nucleolar RNA SNORD27 expression, while transfection of CMV-miR-29b inhibited 88% of carnitine palmitoyltransferase 1b (Cpt1b) expression. Both are not predicted miR-29b target genes, which may suggest that indirect or non-specific effect of miR-29b exist in the cells treated with TGF-β.

## Conclusion and discussion

Our experimental data clearly demonstrated the concept that using a Col1a1 promoter as a sensor produces much less basal expression of miR-29b compared to a conventional overexpression method using a CMV promoter. However, the expression level can be stimulated by TGF-β treatment and thus maintain a dynamic balance between the ECM gene expression and miR-29b. We also showed that through lentivirus, this expression vector can be delivered to animals for potential treatment of tissue fibrosis. It is well established that TGF-β pathway is a potent activator of profibrotic effects in cells and has been described as the “master regulators” of fibrosis [3, 33]. A variety of strategies have been developed to target the TGF-β signaling, including TGF-β receptor inhibitors [34] and monoclonal antibody against TGF-β [35]. However, due to its importance in normal cells, the therapeutic methods that totally block the TGF-β pathway have been shown to cause significant cardiovascular toxicity [3], which include inflammatory infiltration in blood vessels and heart valves [36] and degeneration of heart valve [37]. Our work showed a possibility of strategically inhibiting downstream of TGF-β pathway by a sensor mechanism, which up-regulates the anti-fibrotic molecules in response to fibrotic stimulation. However, it is recognized that the clinical application of gene therapy using lentivirus is still challenging [38–40], but some non-integrating delivery vehicles and techniques are also in development [41]. The purpose of our current study is a proof of concept, and this concept may have a broader application for other disease-related genes and their regulatory molecules.

Strategies to target specific organs, tissues, or cell types are commonly used in drug development to avoid the off-target effect. A recent publication employs engineered immune cells to target the activated fibroblasts that express the fibroblast activation protein, which showed great potential in mouse model to combat cardiac fibrosis [42]. However, tissue fibrosis occurs in multiple organs and involves several types of cells known and unknown [2]. Targeting one cell type may not be sufficient to block tissue fibrosis because multiple types of cells could be involved in the formation of fibrosis. To resolve this issue, we developed this novel strategy to target any cell type that contributes to tissue fibrosis. While the mechanisms of fibrosis formation remain elusive, it has been demonstrated that increased synthesis and secretion of collagen and other extracellular matrix (ECM) proteins into the interstitial space is a common step in fibrosis [2, 13–16]. Therefore, we took advantage of this feature and designed the expression vector to strategically target the cells with elevated collagen synthesis using an exogenous col1a1 promoter as sensor and miR-29b as an effector. With fluorescent protein genes constructed in this vector, it can also be used as a reporter of collagen synthesis and a research tool *in vivo* to identify novel cell populations that participate in fibrosis.

In addition, the miR-29b could be replaced with a more specific siRNA against collagen to reduce off-target effects if desired. However, it is debatable whether targeting one specific gene is superior to targeting multiple related genes. It has been recognized that small changes in related genes within a specific pathway may have more significant biological effect even if they are not statistically significant for each gene [31, 43]. Based on our RNA-sequencing data, miR-29b overexpression targeted a broad spectrum of fibrosis-related genes, but the inhibition is mostly moderate. In the RNA-sequencing analysis, we found a very interesting gene expression pattern in the TGF-β treated cells, and pathway analysis showed that the top hit pathways are closely related to fibrosis. We believe that the pathway analysis including all gene expression changes provides more systematic information about the biological perturbation compared to just focusing on individual gene expression.

We also examined the miR-29b targeting genes in these experiments using RNA-sequencing data. Hundreds of genes are predicted to be the targets of miR-29b with different scores listed in the miRDB database (http://www.mirdb.org/). However, most of these target genes have not been experimentally verified. Our data showed that about one-third of the top 100 predicted target genes were not downregulated by overexpression of miR-29b, some were even upregulated. It is interesting to note that miR-29b reverses several ECM gene expression, including different isoforms of collagen even though the reversal effect on each gene was not dramatic, nonetheless, the anti-fibrotic effect of miR-29 family microRNAs has been convincingly demonstrated [18–23, 25–27]. It is also important to note that even though the change in mRNA level is moderate, the change in protein level could be more significant due to accumulative effect, e.g. the Western blot data in Fig 6 showed more than 50% decrease after miR-29b overexpression in TGF-β treated cells.

## Supporting information

S1 File (PDF)

## Abbreviations

CMV: cytomegalovirus
Col1a1: collagen 1a1
DMEM: Dulbecco's modified Eagle's medium
DEG: differentially expressed gene
ECM: extracellular matrix
KEGG: Kyoto Encyclopedia of Genes and Genomes
MEF: Mouse Embryonic Fibroblasts
MI: Myocardial infarction
miR: microRNA
