# Supplementary material for: A strategic expression method of miR-29b and its anti-fibrotic effect based on RNA-sequencing analysis
Source: PLoS One. 2020 Dec 17;15(12):e0244065. doi: 10.1371/journal.pone.0244065 (PMC7746150; doi:10.1371/journal.pone.0244065)
Supplement: S1 File — (PDF) [file pone.0244065.s001.pdf]

Support Information

This Support Information include the Uncropped and Unadjusted Western Blot images.

Original Western Blot for Fig 6A and Fig 6B.

Collagen I

GAPDH

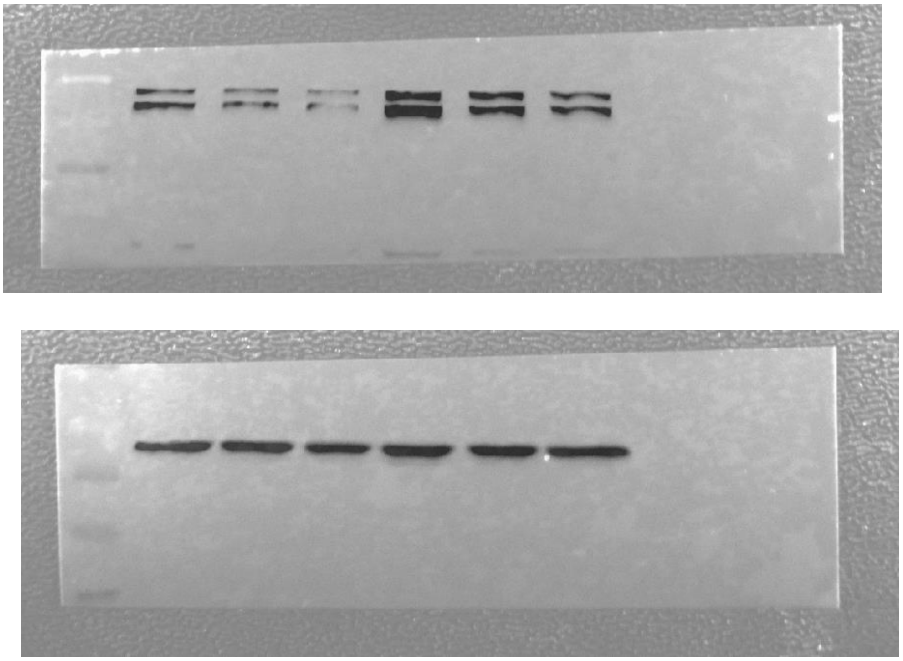

Fig 6A Full Western Blot

Collagen I

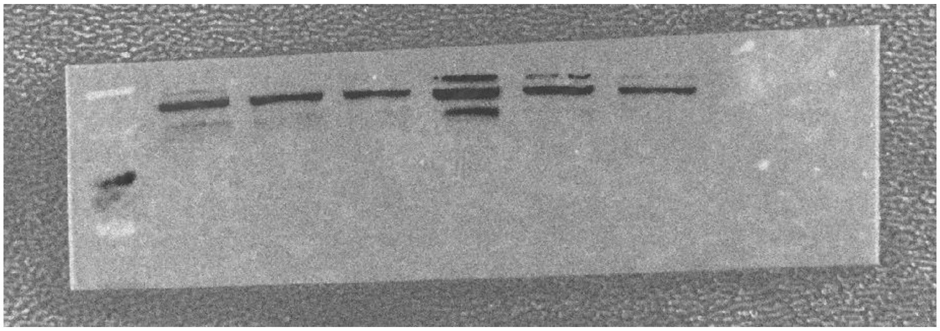

Fig 6B Full Western Blot
